# Protein Folding and Misfolding on Surfaces

**DOI:** 10.3390/ijms9122515

**Published:** 2008-12-09

**Authors:** Massimo Stefani

**Affiliations:** Department of Biochemical Sciences and Research Centre on the Molecular Basis of Neurodegeneration (CIMN), University of Florence, Florence, Italy. E-Mail: stefani@scibio.unifi.it; Tel. +39-055-4398307; Fax: +39-055-4398905

**Keywords:** Protein folding, protein misfolding, protein aggregation, amyloid, amyloid fibrils, amyloid cytotoxicity

## Abstract

Protein folding, misfolding and aggregation, as well as the way misfolded and aggregated proteins affects cell viability are emerging as key themes in molecular and structural biology and in molecular medicine. Recent advances in the knowledge of the biophysical basis of protein folding have led to propose the energy landscape theory which provides a consistent framework to better understand how a protein folds rapidly and efficiently to the compact, biologically active structure. The increased knowledge on protein folding has highlighted its strict relation to protein misfolding and aggregation, either process being in close competition with the other, both relying on the same physicochemical basis. The theory has also provided information to better understand the structural and environmental factors affecting protein folding resulting in protein misfolding and aggregation into ordered or disordered polymeric assemblies. Among these, particular importance is given to the effects of surfaces. The latter, in some cases make possible rapid and efficient protein folding but most often recruit proteins/peptides increasing their local concentration thus favouring misfolding and accelerating the rate of nucleation. It is also emerging that surfaces can modify the path of protein misfolding and aggregation generating oligomers and polymers structurally different from those arising in the bulk solution and endowed with different physical properties and cytotoxicities.

## 1. Introduction

Increasing interest of protein science researchers is currently focussed at unravelling the molecular basis of protein folding and of its counterpart, protein misfolding and aggregation, as well as of the mechanisms of aggregate toxicity to living systems. The intense efforts spent in the past years to gain knowledge in these topics arise mainly from the awareness that it can shed light on the “folding code” built in most natural polypeptide chains as well as on the biochemical basis of a number of degenerative conditions that, at least in some cases, are of dramatic social impact [1–4]. The latter are a group of protein deposition pathologies including neurodegenerative diseases such as Alzheimer’s, Parkinson’s and prion diseases, several systemic amyloidoses, type 2 diabetes, and others. Such diseases are characterized by the presence, in the affected tissues, of proteinaceous intracellular inclusions or extracellular deposits (amyloid plaques) whose main components are fibrillar aggregates arising from the polymerization of one out of around 30 proteins or peptides, each specific of a particular disease, that share basic structural features. Currently, it is believed that the aggregated material is the cause, rather than an epiphenomenon, of the clinical symptoms of the differing amyloidoses and that the latter, at least in the case of the neurodegenerative diseases, can ultimately be traced back to the cytotoxic effects of the aggregates [1–4].

It is generally believed that protein folding and protein aggregation into amyloids are competing pathways relying on the same physicochemical principles (see later). Therefore, any increase of the information on the molecular features of protein folding can be useful to improve our knowledge on protein misfolding and aggregation and vice versa. Presently, it is widely accepted the idea that, similarly to protein folding, even the ability to polymerize into amyloid assemblies is a generic property of polypeptide chains inherently built in their peptide backbone and that its basic principles are shared by all aggregating peptides/proteins [4]. In particular, general consensus has been reached on the idea that protein/peptide aggregation starts from unstable, misfolded/unfolded states that initially assemble into disordered globular oligomers subsequently undergoing reorganization into beta-sheet-rich assemblies growing into ribbons and eventually into ordered fibrils [5]. However, recently, protein aggregation into fibrillar structures from natively folded states has also been reported (see below).

**Figure 1.**
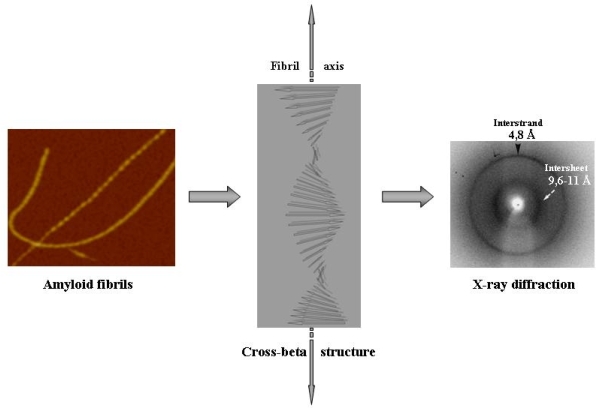
Outline of amyloid fibril structure. Amyloid fibrils are typically formed by two or more elementary protofilaments twisted around each other in a rope-like structure (left, AFM image). Each protofilament displays a shared ordered core structure where each monomer provides beta strands that stack in register forming a double parallel or antiparallel twisted beta sheet that propagates along the major axis of the fibrils whose beta strands run perpendicular to the fibril main axis (what is known as cross-beta structure, centre image). The X-ray diffraction signature of such structure (right image) are the reflections at 4,8 and 9,6–11 Å provided by the regular distance between the stacked beta strands and the sheets, respectively.

Despite the large differences in the structures of the proteins and peptides aggregated in the assemblies found in the differing amyloidoses or produced *in vitro*, amyloid fibrils share basic structural features and an ordered core structure. A typical amyloid fibril is straight, unbranched, 6–12 nm in width and reaches around 1 μm in length; it is formed by a variable number of elementary filaments (protofilaments) twisted around each other, typically 1.5–2.0 nm in diameter [6,7]. The main structural hallmark of amyloid fibrils is the ordered core of their protofilaments. The latter results from a double beta sheet which extends along the main filament axis and whose beta strands provided by each monomer stacks in register and run perpendicular to the fibril axis (what is known as cross-beta structure, whose signatures are the reflections at 4,8 and 9,6–11 Å in X-ray diffraction patterns) [8,9] (Figure 1). The recently described occurrence of amyloid fibrils with biological functions in several microbial and mammalian systems can lead to consider the amyloid fold among those occurring in the functional proteins rather than an aberrant protein structure [10].

Increasing consensus has been reached in recent years on the identification of the most cytotoxic amyloids. Accordingly, the heterogeneous population of unstable fibrils precursors collectively known as protofibrils, or prefibrillar aggregates, are considered the most highly cytotoxic assemblies whereas amyloid fibrils are presently considered stable, relatively inert and harmless benign structures able to recruit the most toxic oligomers [4, 11–16]. Fibril precursor toxicity was confirmed by several findings showing that the appearance in tissue of small toxic oligomers precedes the expression of the clinical phenotype; this could explain the frequently observed lack of relationship between the load of fibrillar amyloid deposits and the severity of the clinical symptoms [17, 18]. These considerations have raised considerable interest on the structural features of these toxic intermediates, although their intrinsic instability and disorder make very difficult their structural characterization.

The potential, as well as molecular mechanism of amyloid fibril nucleation and growth can be different in differing peptides and proteins as well as under varying medium conditions; however, in general, the onset of the process is triggered by any factor increasing the concentration of conformational states with high aggregation propensities, usually misfolded/unfolded species [4]. These conditions can favour thermodynamically protein aggregation, notably by reducing the conformational stability of the protein, as it can be the case of mutations, environmental changes or chemical modifications. Other factors can favour kinetically amyloid nucleation; this is the case of any increase of the amount of the protein/peptide following increased production, reduced clearance, or as a result of mutations with no effect on protein stability but able to increase the rate of oligomerization of the unfolded or partly folded monomers into aggregation nuclei (see later).

Protein folding, but also misfolding and aggregation, in the biological environment can be differently affected by conditions such as the presence of high macromolecular concentrations or highly reactive molecules including reactive oxygen species and sugars that promote destabilising chemical modifications of proteins (oxidation, glycation). Moreover, the biological systems are characterised by the presence of extended surfaces. The latter include those specifically provided by the molecular chaperones favouring protein folding and others such as those provided by membranes and macromolecules. Actually, a natively folded protein or an unfolded peptide can undergo misfolding and structural reorganization with aggregate nucleation in the presence of suitable surfaces favouring non-native conformations with increasing tendency to aggregate (see later).

Protein folding is subjected to a very intricate and precise quality control including the ER and cytosolic molecular chaperones, the ER membrane carriers performing the retrograde transport of proteins unable to fold in the ER lumen [19], the ATP-dependent proteolytic complexes in mitochondria [20], the components of the ubiquitin-proteasome pathway and two complex physiological responses such as the unfolded protein response and the heat shock response (reviewed in [21]). These mechanisms are aimed at hindering the appearance, in the cell, of any misfolded state potentially able to generate aggregation nuclei [4, 21] and are particularly efficient leading to degradation of around 30% of newly synthesized proteins [22]. Accordingly, protein aggregation can be favoured by the impairment or overwhelming of any of these molecular machineries.

This review will present some of the most recent finding and ideas on some of the basic features of protein folding, misfolding and aggregation with special focus on the role performed by inorganic, organic and biological surfaces.

## 2. Essentials of protein folding

The protein folding problem has been a challenging issue shrouded in mystery for decades, until the development of the energy landscape theory. Actually it is not easy to understand how a polypeptide chain with an astronomical number of allowed conformations in its unfolded state is able to point to, and to reach with high precision, the native compactly folded structure in time lengths as short as milliseconds or fractions of seconds.

If we take a simple 100 residue polypeptide chain and assume that each residue has only two allowed conformations, it results an overall 2^100^, or 10^30^, possible conformations of the whole molecule; this means that, at the concentrations used in a test tube folding experiment (i.e. around 10^18^ molecules) and the persistence times given by the molecular dynamics at room temperature (10^11^ s^−1^) each molecule in a given instant will exhibit a conformation different from that of any other molecule in the population. Yet, such an astonishing structural heterogeneity folds in very short times into a peculiar conformation endowed with a minimal conformational energy.

**Figure 2.**
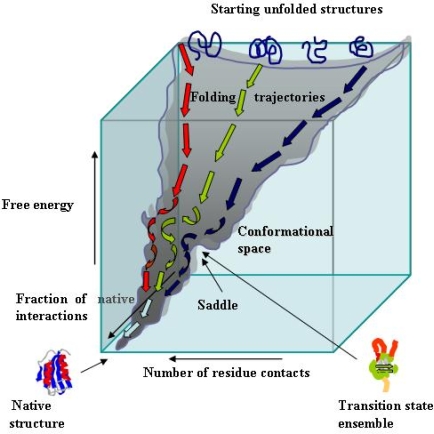
Folding energy landscape. A schematic folding energy landscape displaying the evolution on a rather crude energy scale of a small protein from its unfolded ensemble (top) to the peculiar structure of the native state (bottom). The funnel-shaped graph biased toward the native state structure is a highly simplified model that recapitulates the changes in parameters such as the available conformational space of the folding molecule in terms of allowed residue contacts (entropy), the number of native contacts and the energy of the conformational ensembles. As the folding proceeds, there is a very rapid cooperative increase of the native contacts together with a reduction of either the conformational space that can be sampled for further contacts and the energy content. The surface funnels the highly heterogeneous multitude of the unfolded conformations to the unique natively folded structure. The saddle region of the surface is a key point in this simple energy landscape corresponding to the transition state ensemble, the fraction of the molecules with the energy needed to cross the folding barrier. The transition state ensemble harbours molecular populations in which specific residues have established the key native-like contacts which determine the overall topology found in the native fold. For further details see under the text.

The natively folded state of a protein cannot be reached by a systematic search among all the possible conformations accessible to its polypeptide chain, that would take an astronomical time, as stated by the Levinthal paradox [23]. Presently, there is general consensus on the view that the “folding code” of a polypeptide chain is entirely built into its amino acid sequence; therefore, the sequences of the natural proteins must have been shaped by natural selection in order to display some specific features exploiting the rapid molecular dynamics that allow specific residues, even those widely separated in the amino acid sequence, to come rapidly and reversibly in close contact with one another. The dynamics of the polypeptide chain of a natural protein in solution allow it to establish continuously native and non-native intramolecular contacts; however, the former are, on average, more stable, and hence more persistent. In addition, the establishment of those contacts is a cooperative process, meaning that these contacts are not mutually elusive but, rather, the conformational limitations provided by one of them favour other native contacts in a self-reinforcing process. Some of the native contacts organize, in times of the order of μs, key sites where elements of secondary structure are formed in a rudimentary native-like architecture and around which other contacts and elements of secondary structure are built. Finally, many experimental data on protein folding *in vitro* have shown that only a few contacts are enough to establish the “folding nucleus” of a protein; the latter primes the overall native topology of the condensed, still highly dynamic molten globule states, of the subsequent ensemble of the energy-rich but entropically restrained rate-limiting transition states (saddle points in the energy landscape, Figure 2) and, when present, of the folding intermediates. It can be concluded that a polypeptide chain finds its lowest energy structure by a trial and error stochastic process that samples only a very reduced conformational space and hence only a very small number of allowed conformations [24].

The energetics of the folding behaviour of a polypeptide chain is best described by the energy landscape of an amino acid polymer. The energy landscape contains all conformational states accessible to the polypeptide chain together with their entropy, free energy and fraction of native contacts (Figure 2). These species are heterogeneous, highly dynamic and disordered conformational ensembles whose structures are far from the native one. The protein energy landscape is encoded in the amino acid sequence and, in the case of natural proteins, is robust (i.e. is not significantly affected by chemical modifications such as amino acid substitutions) and has the very special appearance of a rough funnel biased towards the native state [24,25]. Although it is not known yet how an amino acid sequence encodes its folding features, it is proposed that the key signature for a protein to fold correctly can be traced back to the pattern of hydrophobic and polar (notably charged) residues that favours preferential interactions of specific residues as the compactness of the structure progressively increases. Once these interactions have established the correct topology of the folding protein, the process evolves rapidly and invariably to generate the native compactly folded structure. Hence, for a polypeptide chain to fold efficiently into a specific three-dimensional structure, a suitable content and a specific pattern of hydrophobic and charged residues must be present. This is confirmed by the analysis of the natively unfolded proteins (NUPs) (see later), a set of natural proteins whose amino acid composition, providing a low mean hydrophobicity and a high net charge, makes them unable to fold in the intracellular environment [26].

Besides ensuring rapidity and precision, the above described behaviour acts as a folding quality control avoiding protein folding into altered states (misfolding) [25]. In fact, when, for some reason, the folding promoting interactions are not formed, the polypeptide chain fails to fold correctly and remains substantially unfolded thus avoiding that partially folded/misfolded structures can be generated.

In conclusion, the current view describes natural proteins as a set of evolved amino acid polymers shaped by natural evolution in order to display secondary structure propensity and other physicochemical features such as mean hydrophobicity and net charge, as well as an overall energy folding landscape suitable to allow them to reach the unique compactly folded native structure efficiently and very rapidly avoiding the appearance of kinetic traps, energetically favoured non-natively structured states that are set off-pathway of the folding process. The energy landscape theory, or “new view” of protein folding, based on statistical mechanics and polymer dynamics rather than on classic chemical dynamics, provides a conceptual framework to describe the general mechanism of protein folding and the rationale to design protein folding experiments and to interpret their results.

## 3. Protein folding on surfaces

Most of the present knowledge on protein folding arises from a multi-disciplinary approach including the use of a variety of simulation and experimental measurements carried out on wild-type and side-directed mutants of the investigated proteins. The experimental tools most widely used range from spectroscopic (CD, DLS, NMR), fluorimetric (fluorescence spectroscopy, FRET), diffraction (small angle X-ray diffraction scattering), mass spectrometry (MALDI-TOF coupled to limited proteolysis) and mutational (protein engineering) techniques to theoretical studies including molecular dynamics simulation and model building. However, these results, although of immense value, in general have been obtained in test tube experiments where a very simple environment was present (usually a buffer with a defined pH and ionic strength containing some co-solvent or denaturing agent). These conditions are very different from those found in the intracellular medium where protein folding takes place and lack the presence of other factors potentially affecting protein folding, misfolding and aggregation.

The key signature of the intracellular milieu is its very high macromolecular concentration, averaging 300-400 mg/mL. This feature, often referred to as macromolecular crowding, has very important consequences in terms of thermodynamics affecting the conformational states of proteins [27]. A very high macromolecular concentration means that the volume freely available to a molecule is only a fraction of the total volume where the macromolecule is dissolved; therefore, the resulting excluded volume effects can favour thermodynamically compact states including both natively folded and aggregated states of proteins, though to a different extent depending on the protein. On this aspect, it has been calculated that an increase in macromolecular crowding from 30% to 33% (in terms of the volume of a given space occupied by molecules) could result in a rise of the molecular binding affinities by as much as one order of magnitude [27]. Such effects can also favour aggregate nucleation when proteins and peptides are unable to fold efficiently into monomeric compact states, although the increased viscosity of the medium can lower the diffusion-limited growth rate of the aggregation nuclei by reducing the translational movements of the macromolecules.

One of the consequences of the intracellular macromolecular crowding is the presence, inside a cell, of a very large surface area including the overall macromolecular surface and that provided by the cell membranes. The latter can favour reversible unfolding/refolding of specific proteins when they physiologically translocate across the membrane [28]; some of the macromolecules found inside a cell can also favour protein folding be providing suitable surfaces. This is the case of the large and heterogeneous family of the molecular chaperones, including the prokaryotic GroES/GroEL and DnaK/DnaJ systems as well as many eukaryotic assemblies such as the Hsp70/Hsp40 system, other Hsp proteins and the crystallins. The role of these molecular chaperones is to provide a suitable environment, in most cases a surface, where a protein can fold rapidly and efficiently avoiding inappropriate interactions without being provided with any further information needed to find its native fold [29]. Actually, in most cases the molecular chaperones are able to specifically recognize exposed highly aggregation-prone segments avoiding their inappropriate interactions with other cellular components or with similar segments exposed on other protein molecules that could possibly result in aggregate nucleation. A recent survey in the protein data bank has shown that these highly aggregating stretches are most often capped by basic residues; such a feature, besides hindering the mutual interaction of these stretches that could enhance the generation of aggregation nuclei, also favours their recognition by molecular chaperones featuring co-evolution of the latter together with some structural features of proteins aimed at hindering their aggregation [30].

Another example of surfaces favouring protein folding is provided by the large family of the above mentioned NUPs [26]. These apparently structurally and functionally unrelated proteins include many transcription factors, ribosomal proteins and signalling proteins involved in the cell cycle control at the transcriptional and translational levels. Unstructured domains are also found in certain regions of other proteins that are otherwise natively folded. A recent search in the Swiss Protein Database has led to the prediction that over 15,000 proteins could contain disordered regions of at least 40 consecutive residues and over 1000 proteins could be completely disordered [31]. This observation indicates that significant segments of the eukaryotic genomes encode long stretches of amino acid residues that, at least under some conditions, are likely to be unfolded or to adopt non-globular structures of unknown nature.

NUPs are usually easily recognizable from their amino acid content as they generally display a low mean hydrophobicity and a high net charge. These characteristics, thought to be the molecular basis by which these proteins remain unfolded in the absence of partners, are also able to reduce their intrinsic tendency to aggregate in the highly crowded intracellular milieu [32]. Here, the unstructured state of most NUPs favours their binding to the molecular chaperones during their short living time before they interact with their specific target proteins [26]. Actually, many NUPs adopt specific three-dimensional structures upon interaction with their specific target proteins that are thought to provide them a surface suitable to allow their folding [34]. It is also possible that the target protein provides some structural information needed for the specific NUP to reach its correct fold, in particular charged and hydrophobic residues complementing its structural deficiencies Alternatively, NUPs undergo rapid intracellular turnover by the cellular clearance mechanisms [33]. The latter feature could be an advantage for certain cellular functions, providing a further level of control to enable the cell to respond rapidly and effectively to perturbations in the cellular environment.

## 4. Protein folding and aggregation are competing pathways

**Figure 3.**
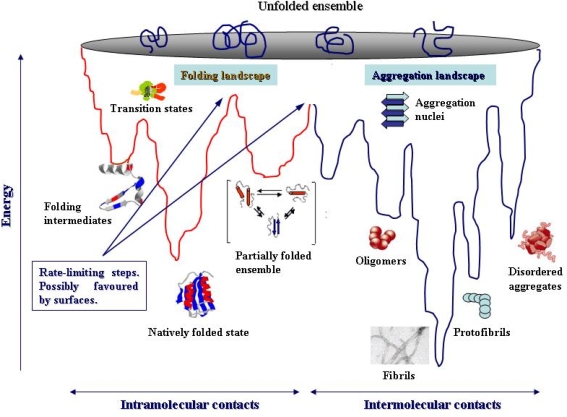
Combined protein folding/aggregation landscapes. A combined energy landscape model for protein folding (left) and aggregation (right) starting from the unfolded ensemble. Both sides display considerable roughness, but amyloid fibrils display a remarkably higher stability and lower energy content than the natively folded structure. The picture highlights the multitude of the different conformational states available to a protein when they are stabilized by either intramolecular (monomeric protein) or intermolecular (aggregation intermediates and mature fibrils) contacts. The presence of intermolecular contacts increases dramatically the ruggedness of the landscape for protein aggregation with respect to what is shown in the folding side. The picture highlights energy barriers that a monomeric polypeptide chain either unfolded or natively folded must overcome to gain access to the aggregation landscape generating aggregation nuclei, often the rate-limiting step of the aggregation process. The energy barriers can be lowered by the presence of suitable surfaces. For further details see under the text. (Modified from [40]).

Until 1998 it was commonly believed that the ability to polymerize into ordered fibrillar aggregates of amyloid type was a shared property of the few proteins and peptides found aggregated in tissue in the various amyloid diseases possibly arising from some structural peculiarity. However, in 1998 it was found that two proteins unrelated to any amyloid pathology were able to aggregate *in vitro* into fibrillar assemblies undistinguishable from the classical amyloid fibrils [35,36]. Since then, it has increasingly been recognized that the tendency to aggregate into amyloid assemblies is a general property of the peptide backbone of proteins and peptides. Such a tendency arises from the primordial tendency of polypeptide chains to self-organize into polymeric assemblies stabilized by hydrogen bonds between parallel or anti-parallel polypeptide stretches in the beta-strand conformation provided by the monomers. The resulting polymers display the ordered cross-beta structure that characterizes the amyloid fold (reviewed in [4]). This does not imply that the side chains of the polypeptide chain are not important; rather, they determine the environmental conditions under which the polypeptide chain can undergo aggregation. This view considers natural proteins as a group of evolved amino acid polymers whose the amino acid sequences disfavour aggregation whilst favouring folding into compact states resulting mainly from the tertiary interactions among the side chains that shield the peptide backbone. Conversely, protein aggregation into amyloid, which is mainly stabilized by secondary interactions, is considered the expression of the intrinsic primordial tendency of the peptide backbone to give secondary intermolecular interactions between backbone groups (reviewed in [4]). Another consequence of such a paradigm is that protein folding and protein aggregation must be distinct but competing pathways the same polypeptide chain can undergo depending on the environmental conditions (Figure 3). Accordingly, extensive studies have been carried out *in vitro* to investigate the transition between natively folded states and soluble aggregate-precursor states and between the latter and mature amyloid fibrils [37].

As it has been pointed out above, the intracellular macromolecular milieu is likely to favour compact states such as those arising from protein folding or protein aggregation. Moreover, protein folding and protein aggregation rely on similar physicochemical parameters of the polypeptide chain including a significant propensity to gain secondary structure, a low net charge, and a relatively high content of hydrophobic residues; these considerations are confirmed by the structural adaptations in the NUPs (see above) and by the data indicating that mutations increasing the mean hydrophobicity or the propensity to generate beta structure or reducing the net charge of any protein/peptide can accelerate its aggregation from an unfolded state [38]. These findings confirm that protein folding and protein aggregation are pathways in close competition to each other and that any polypeptide chain can undergo either pathway depending on both its structural and physicochemical features and medium conditions.

The view that protein folding and aggregation are competing paths considers both as distinct yet not mutually excluding processes relying on a more general energy landscape including conformational states not involved in protein folding, yet potentially accessible to a polypeptide chain [39]. The two sides of the protein energy landscape highlight the competition between intramolecular (folding) and intermolecular (aggregation) interactions, which increases considerably the roughness of the whole landscape. The scheme depicted in Figure 3 (modified from [40]) suggests, at least in part, the complexity of the overall protein folding and aggregation energy landscape. It includes some of the main conformational states a polypeptide chain can get during its self-organization paths eventually culminating with the appearance of thermodynamically favoured compact monomeric or polymeric states [39]. Either stable final compact state may be even more favoured thermodynamically in a living cell by the macromolecular crowding and its excluded volume effects (see above).

The generation of oligomeric aggregation nuclei is considered a key step at the onset of protein aggregation, accounting for the delay times of polymer appearance that are recorded by in *in vitro* protein aggregation experiments. However, at variance with protein folding, where in depth investigations carried out in the last decade have provided significant information on the structural features of folding intermediates and transition states, much less knowledge is currently available on the conformational states available to an aggregating polypeptide chain; the structural features, at the atomic level, of the oligomeric assemblies arising in the path of protein aggregation are substantially unknown as well. Extensive investigation on alpha-synuclein has shown that, in this case, the transient oligomeric species are rich in beta-sheet, expose hydrophobic clusters and display a partially folded structure [40, 41]. Actually, some of the energy minima in the aggregation side of the energy landscape are expected to be poorly defined due to the broad heterogeneity of unstable, rapidly interconverting oligomeric states endowed with comparable free energies. On the contrary, the energy minima of the much more structurally defined stable higher order species (protofibrils, protofilaments and mature fibrils) can be much more easily identifiable, even though fibrils with different morphologies and structural differences can be formed under different solution conditions [42]. For example, the stabilities, and hence the energy minima, of mature amyloid fibrils, and their structural variants, are expected to be more pronounced than those of their natively folded monomers considering the reduced molecular dynamics and the consistency of the ordered core structure of the fibrils. The nucleation-dependent polymerization mechanism of fibril growth, whose physical basis approaches that of the ordered assembly occurring in crystal growth, also supports fibril stability and represents a key difference between protein folding and aggregation.

It is possible to shift a protein from the folding to the aggregation side of its energy landscape by modifying its structural features (mutations, truncations, amino acid chemical modifications) or the environmental conditions (temperature, pH, medium composition). Increasing the concentration of the specific protein/peptide can also result in aggregate nucleation when the level of the nucleation precursors exceeds a critical concentration (reviewed in [4]). In most cases, protein aggregation can be started in the presence of mildly destabilising medium conditions, such as mild shifts of the temperature or the pH or the presence of moderate amounts of denaturing agents or of co-solvents such as trifluoroethanol; the latter modifies the dielectric constant of the solution increasing the stability of the secondary contacts while reducing that of the tertiary ones [43–45]. Under these conditions, a folded protein populates partially unfolded states by opening its closely packed structure, thus exposing aggregation-prone regions normally buried into the hydrophobic core and the peptide backbone, that is shielded by the side chains in the compactly folded state. These partially unfolded structures can bear similarities to the folding intermediates [38, 46, 47] or to some of the near-native conformations in dynamic equilibrium with a folded protein. The link between native state dynamics and fibrillar aggregation of a protein has been highlighted in the case of lysozyme by mass spectrometry experiments [48]. In the lysozyme, the relative instability of the partially folded precursors is the driving force allowing them to re-organize into still poorly stable, and often thermodynamically disfavoured, transient aggregation nuclei rich in beta structure, established between stretches of polypeptide chains in the beta strand conformation [48].

Nucleation is the rate limiting step of the aggregation process and occurs during the lag phase; its kinetics can depend on the protein and medium conditions, whereas subsequent nuclei elongation is thermodynamically favourable and proceeds until completion of fibril assembly. Spherical oligomers and other pre-fibrillar forms, including curvy protofibrils, can be formed instead of aggregation nuclei and appear to result from a nucleation-independent path in the absence of any lag phase [49–52]. In this case, it is not clear whether these forms are on-pathway, growing by direct binding of monomers, or off-pathway, representing dead-end intermediates [51, 53–55]. Studies on beta-2-microglobulin (b2-m) have provided information on this issue. It has been shown that, depending on protein structural features and medium conditions, b2-m exists in different aggregation states; for b2-m and other proteins, some of these states (oligomeric species and beaded protofibrils) are off-pathway products [42,51,56] arising from the polymerization of partially folded species retaining significant amount of native structure and involving some of the native beta strands [57]. The latter species are different from the oligomers appearing in the fibrillization path, which involve extensive structural rearrangement into the stable cross-beta structure of amyloid fibrils [52].

Almost no information is currently available on the energy barriers a folded or a partially unfolded protein must overcome to gain access to the conformational spaces allowing it to re-organize into aggregation nuclei; however, it is believed that these structural transitions can be favoured, among others, by surfaces (Figure 3). This is a key issue, considering that the intracellular environment provides an extremely large surface area. Actually, surfaces, either biological or synthetic, can enhance protein misfolding and speed aggregation (see below) besides favouring protein folding in special cases (see above).

**Figure 4.**
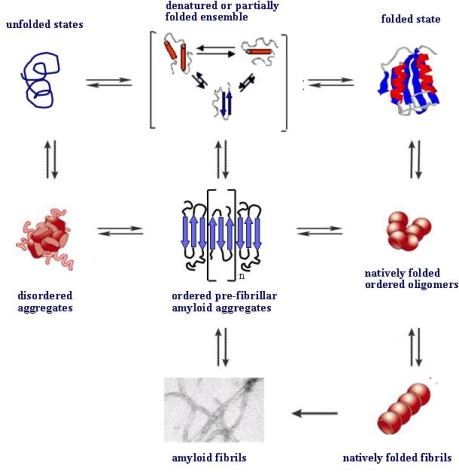
Aggregate nucleation from unfolded or natively folded states. The nucleation of oligomeric pre-fibrillar aggregates is the key, as well as the rate-limiting, step in the path eventually culminating with amyloid fibril formation. Usually, the process is initiated by the misfolded fraction of protein/peptide molecules either in equilibrium with the compactly natively folded states or arising from protein destabilisation following mutations, chemical modifications or any alteration of the physicochemical features of the environment. Recently, it has been reported that, in some cases, protein aggregation can start from natively folded states organising into oligomers. The latter can further grow into native-like fibrils, as in the case of serpins or Ure2p or, alternatively undergo conformational rearrangement populating misfolded pre-fibrillar aggregates. For further details see under the text.

Finally, recent findings have shown that, at least in some cases, a protein can aggregate by initially populating monomeric or oligomeric states where it maintains substantially its natively folded structure before subsequently undergoing conformational rearrangements into amyloids (Figure 4). In addition to the natively folded beaded protofibrils of b2-m (see above), other proteins have been proposed to undergo ordered fibrillar polymerization retaining their native fold at least in the initial steps. These include tranthyretin, for which a model with direct stacking of natively folded monomeric subunits has been proposed [58], T7 endonuclease I [59], p13suc1 [60], and the serpins (reviewed in [61]), where a domain swapping mechanism has been depicted. A similar mechanism could also underlie the generation of native-like fibrils by the yeast prion Ure2p [62] and the first step of the amyloid aggregation of the Sso acylphosphatase [63].

## 5. Surfaces can favour protein unfolding/misfolding

**Figure 5.**
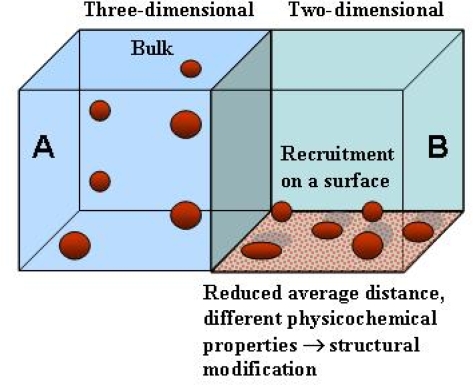
Distribution of molecules in a 3D or a 2D space. Distribution of the molecules in a three-dimensional (**A**) or in a two-dimensional (**B**) space such as those provided by the bulk solution or by a surface, respectively. In a 3D space, the average distance between protein molecules is in relation to the cube root of the total number of molecules whereas in a 2D space this depends on the square root of the number of molecules. As a consequence, for concentrations above 1 nM the same number of protein molecules typically are much closer to each other in a two-dimensional space than in the corresponding three dimensional space. Therefore, surfaces can locally increase protein concentration favouring reciprocal interactions and speeding aggregate nucleation. For further details see under the text.

As stated above, proteins are synthesized and fold in a very complex environment where they are in close contact with other molecules and with biological surfaces such as membranes and macromolecular assemblies favouring, in some cases, their correct folding. However, biological surfaces, notably lipid membranes, can also affect the conformation of the interacting proteins populating secondary structure-based aberrant states of the polypeptide chain [64,65] thus modifying lipid arrangement with possible membrane disruption [66,67]. Surfaces can also recruit protein molecules increasing their local concentration and/or their proximity to each other in a two-dimensional environment (Figure 5). Both effects can result in enhanced tendency of proteins/peptides to undergo aggregation. Actually, pre-fibrillar assemblies can grow on nanoparticles [68], anionic surfaces such as mica, fatty acid and SDS micelles, and anionic phospholipid vesicles [69–71], synthetic phospholipid bilayers [72–78] and cell membranes [79,80], modifying membrane structure and permeability and impairing the function of specific membrane-bound proteins and signalling pathways [81,82]. These studies carried out mainly with synthetic surfaces, have prompted increasing interest on the role of surfaces in protein aggregation and on the relation of the latter to the membrane structure and lipid composition.

The effects of a surface on protein misfolding and aggregation depend on the chemical features of the monomer, its folded or unfolded state, the way it interacts with the surface and the physicochemical properties of the latter, including its electrostatic potential and hydrophobicity (reviewed in [83]). In the case of lipid membranes, density of lipid packing, curvature, compactness, rigidity or fluidity can also be important in affecting the features of monomer/oligomer interaction. The physicochemical properties of the two-dimensional environment of a surface can be very different from those of the bulk aqueous phase. For monomer concentrations above 1 nM, surface adsorption reduces considerably the average distance among molecules respect to that in the three-dimensional bulk solution favouring monomer-monomer interactions, aggregate nucleation and insertion into the lipid bilayer [69, 73–78] (reviewed in [84, 85]) (Figure 5), eventually leading to membrane disorganization [65, 67]. In addition, the strong electrostatic field or the non-polar environment of heavily charged or hydrophobic surfaces, respectively, can modify the protein fold with exposure to the surface of regions that normally are associated with each other through electrostatic or hydrophobic interactions [85]. This view agrees with experimental data showing that surfaces can catalyze the formation of amyloid aggregates by a mechanism substantially different from that occurring in the bulk solution [69].

In the adsorbed state, proteins/peptides are at the interface of two phases with different physicochemical properties, and protein residues are involved in interactions with surface-exposed functional groups that can favour non-native structural states. In particular, hydrophobic or charged surfaces may induce local or more extensive unfolding resulting in the opening of the closely packed structure; concomitantly, hydrophobic groups normally buried into the compactly folded native state are allowed to interact with hydrophobic clusters exposed on the surface without paying the energy penalty resulting from the exposure of the same residues to the aqueous environment (reviewed in [85]). As discussed above, these considerations apply to the behaviour of chaperones in assisting protein folding, to the target-induced folding of natively unfolded proteins as well as to the trafficking of protein molecules across membranes. In most cases, the interaction of a misfolded or unfolded species with a lipid membrane is likely to occur via a two-step mechanism involving the electrostatic interaction of the positively charged residues with negatively charged or polar lipid head groups with structural alteration; this is followed by the insertion of hydrophobic regions of the protein/peptide inside the bilayer (reviewed in [86]) where, in general, the hydrophobic interior favours structural changes and secondary interactions resulting in enhanced tendency of proteins and peptides to aggregate, as it has been shown for the prion protein, the Abeta peptides and amylin [64–66, 87].

However, not only membrane surfaces can be involved in protein aggregation. For example, it has been proposed that b2-m aggregation can be favoured by monomer binding to the collagen triple helix, thus providing a possible explanation of the tissue-specificity of dialysis-related amyloidosis [88]; it has also been proposed that binding affinity fluctuations could influence the concentration of wild-type and N-truncated b2-m in the proximity of collagen fibers and hence their susceptibility to aggregation [89]. Finally, recent findings suggest that, in the presence of collagen, monomeric b2-m aggregates into amyloid fibrils sprouting from the surface of collagen fibres either *in vivo* and *in vitro* [90]. Glycosaminoglycans can also provide a surface suitable to promote growth of amyloid assemblies of gelsolin [91] and acylphosphatase [92]. In addition, other polyanions such as SDS and nucleic acids have been found to accelerate fibrillization of alpha-synuclein and the prion protein, respectively [70, 93]. Finally, as stated above, clusters of anionic phospholipids have been shown to e enhance protein misfolding and aggregation [70–72] and preferentially recruit protein aggregates (see below).

Biological membranes may also be important in amyloid fibrillogenesis as the primary sources of the aggregating peptide monomers. Membrane environment is of fundamental importance in regulating membrane protein degradation by specific membrane proteases such as the secretases or the protein convertases. This is best exemplified by the Abeta peptides resulting from APP processing [94], the ABri and ADan peptides resulting from BRI processing (reviewed in [82]), the medin, and gelsolin peptides arising from lactadherin and gelsolin proteolysis, respectively [95] (reviewed in [96]), as well as other peptides such as that arising from Pmel17 processing (reviewed in [96]).

Conflicting data on the effect of membrane cholesterol on amyloid aggregate production and toxicity have been reported in the past years (reviewed in [97, 98]). On this aspect, the cholesterol-AD relation is paradigmatic (reviewed in [99]). The positive relationship between hypercholesterolemia and risk of sporadic AD is known since long time, however a mechanistic explanation for such association has not yet been provided. Yet, many data suggest a protective effect of membrane cholesterol against aggregate cytotoxicity [100]; in addition, a loss of cholesterol in brain leads to neurodegeneration and reduced levels of cholesterol are found in brains from AD patients [101]. Possible clues on the effect of cholesterol on amyloid generation and interaction at the membrane level can be given by lipid rafts, ganglioside- and cholesterol-enriched dynamic membrane microdomains harbouring many membrane proteins including APP and secretases (reviewed in [102]). The increased presence of APP and secretases in lipid rafts may provide, at least in part, a theoretical framework for the observed increased AD risk in hypercholesterolemic people (reviewed in [99]). On the other hand, conflicting results have highlighted that altered cholesterol content in neuronal membranes could favour the amyloidogenic or the non-amyloidogenic pathway of APP processing with increased or reduced Aβ40/42 production, respectively [103–105]. The generation of amyloidogenic peptides arising from membrane processing of other proteins (reviewed in [83, 96]) could be affected by membrane lipid composition as well.

## 6. Biological surfaces are primary sites of amyloid interaction and toxicity

The question as to whether amyloid fibrils are toxic to cells by themselves or, rather, they are harmless, stable reservoirs of toxic precursors stems from long time. Actually, in some cases mature amyloid fibrils have been reported to impair cell viability [106, 107]; however, it is increasingly recognized that unstable oligomeric assemblies appearing in the path of fibrillization are endowed with the highest toxicity (reviewed in [4]) thus accounting for the lack of direct correlation between density of fibrillar plaques in the brains of Alzheimer’s disease patients and severity of their clinical symptoms [17]. Considering the difficulty to get structural information on the intermediates (protofibrils) preceding the appearance of mature fibrils (see above), much interest has recently been focused on their morphological features, as shown by electron or atomic force microscopy. The earliest protofibrils typically appear as globular assemblies 2.5–5.0 nm in diameter spontaneously organizing into chains and variously sized rings often comprising small “doughnuts” with a central 2–3 nm wide pore (amyloid pores) [11–16]. Such subpopulation of pre-fibrillar ring-shaped aggregates could account for amyloid toxicity, thus envisaging a basically common early biochemical mechanism of the latter through cell membrane permeabilization (reviewed in [86]) (see also below) in a way that resembles the mechanism of several microbial pore-forming toxins [108]. Channels or pores formed by pre-fibrillar amyloid aggregates have been described *in vitro* for a number of peptides and proteins associated or not-associated with amyloid disease (reviewed in [4]) and characterized primarily by recording ion currents across biological or reconstituted membranes [77]. “Doughnuts” or channel-like assemblies of pre-fibrillar aggregates of many peptides and proteins have also been observed by EM and AFM [11, 109–111].

Besides recruiting protein monomers favouring their misfolding and aggregation, surfaces, notably cell membranes, can also bind actively the unstable oligomeric assemblies preceding the appearance of mature amyloid fibrils. The importance of the relation between membrane lipid composition and the ability of early aggregates of peptides and proteins to bind to and to disassemble membranes has been extensively investigated. Many studies highlight the key role of either anionic surfaces and membranes containing anionic phospholipids; as specified above, the strong electrostatic field given by clusters of negative charges can favour protein unfolding and aggregate nucleation; however, clusters of negative charges can also be sites of preferential interaction with pre-fibrillar aggregates. Accordingly, it has been shown that pre-fibrillar assemblies interact with, and destabilise, synthetic phospholipid bilayers [73–76, 78, 112] (reviewed in [86]) and cell membranes [79, 80], modifying membrane permeability and impairing the function of specific membrane-bound proteins and signalling pathways [81]. The roles of cholesterol and gangliosides in modulating Abeta peptide generation and aggregation (see above) as well as membrane-aggregate interaction have also been extensively studied. Actually, it has recently been reported that pre-fibrillar aggregates supplemented to the cell culture media display reduced interaction with the cells and cytotoxicity upon enriching in cholesterol the cell membrane whereas the opposite effects were found in cholesterol-depleted cells [105, 113–115]. Although requiring more extensive research, these data support the idea that, in general, a higher membrane rigidity following increased cholesterol content can hinder aggregate interaction with the cell membranes thus enhancing membrane resistance against disassembly by the aggregates.

Another question debated since long time is whether specific receptors for amyloids responsible of the amyloid-membrane interaction do exist on the cell membrane. The surface of the cell membrane is crowded of protein molecules. It has been estimated that the average plasma membrane surface is around 2,000 μm^2^ with a density of membrane proteins averaging about 20,000 molecules/μm^2^, for a total 40 × 10^6^ protein molecules per cell surface. It is therefore conceivable that amyloid oligomers contacting protein molecules sprouting from the cell membrane may interact more or less specifically with some of them.

In the past, several cell surface proteins have been considered as possible candidate receptors of Aβ aggregates. These receptors could be specific for the shared cross-beta fold rather than for any peculiar structural feature of the Aβ peptides although, in some cases, they could also be monomer-specific, as in the Aβ-APP or Abeta-TNFR1 interactions proposed to be at the origin of Aβ cytotoxicity [116, 117]. Since 1996, the receptor for advanced glycation end products (RAGE) has been proposed as a major candidate as amyloid receptor [118]. RAGE is increased in systemic amyloidoses, is able to interact with amyloid assemblies made from serum amyloid A, amylin and prion-derived peptides [119] and appears involved in Alzheimer’s and Creutzfeldt-Jacob diseases [120, 121]. By competing for ligand binding with cell-surface RAGE, its plasma soluble form, sRAGE, might trap circulating ligands preventing their interaction with cell surface receptors. Actually, sRAGE appears protective against cytotoxicity of transthyretin aggregates [122] and its high plasma levels are associated with a reduced risk of several diseases including AD. Increasing plasma sRAGE is therefore considered a promising therapeutic target potentially preventing vascular damage and neurodegeneration [123].

In addition to RAGE, several cell surface proteins, including voltage-gated [124] or ligand-gated calcium channels such as the glutamate NMDA and AMPA receptors have also been considered as possible receptors or specific interaction sites for amyloids [125–127]. In addition, tissue-type plasminogen activator (tPA) has also been proposed as a multiligand specific for the cross-β structure [128]. Finally, increasing evidence suggests that additional neuronal binding sites could be involved in the interaction with the plasma membrane of amyloid aggregates made from different peptides and proteins [129]; these could include anionic lipid clusters, as suggested by the finding that rising the content of negatively charged lipids results in increased channel formation by amyloids in synthetic lipid bilayers [66] and that annexin-5 protects against Aβ-peptide cytotoxicity by competing at common PS-rich sites [130].

The presence of specific effects mediated through the preferential, or even specific, interaction with membrane proteins could, at least in part, explain the variable vulnerability to amyloids of different cell types [113]. However, in spite of these and other data on specific interaction sites for amyloids, the tendency of early amyloid aggregates to interact with synthetic lipid membranes supports the idea that the interaction can be non-specific but, possibly, modulated by the membrane lipid content (see above); such an interaction can also be able, by itself, to impair cell viability by altering membrane structure and permeability.

Since 1993 it was proposed the “channel hypothesis” of amyloid toxicity, whereby the toxic aggregates form non-specific pore-like channels in the membranes of the exposed cells [73] (reviewed in [86]). The proposal is now supported by studies carried out both on synthetic phospholipid bilayers and on cell membranes showing that the function of specific membrane proteins is impaired by the interaction with misfolded species or their oligomers (reviewed in [86]), [131]. For example, the size-dependent permeabilization of artificial vesicles by protofibrillar α-synuclein suggests that permeabilization may occur mainly as a result of a specific membrane perturbation via the formation of pores at least 2.5 nm in diameter coexisting with fibrils, raising the possibility that, at the conditions found in the cytoplasm, “pores” may be stable enough to be the true pathogenic species in Parkinson’s disease [12]. The ability of most amyloidogenic peptides and proteins to form “pores” in their aggregation path convincingly supports the idea that membrane permeabilization can result even *in vivo* from the presence of such species and be the key trigger of cell sufferance and death (reviewed in [132]). Should the channel hypothesis gain further experimental support and be extended to proteins and peptides associated with other amyloid diseases, then inhibition of “pore” production would represent a solid rationale in the search of molecules to be used in amyloid disease therapy.

The presence of toxic aggregates inside or outside the cells impairs a number of functions ultimately leading to cell death by apoptosis or, less frequently, by necrosis [12, 109, 133–138]. This is true even for aggregates formed from proteins not associated with amyloid disease, featuring cytotoxicity as a generic property of every amyloid aggregate possibly arising from their shared cross-beta structure (reviewed in [4]) [138]. In most cases, initial perturbations of fundamental cellular conditions such as redox status and free Ca^2+^ levels appear to underlie the impairment of cell function induced by the aggregates [15, 110, 137, 139–144].

In general, intracellular oxidative stress in cells exposed to toxic aggregates has been related to some form of destabilisation of cell membranes resulting in the lack of appropriate regulation of membrane proteins such as specific enzymes, receptors and ion pumps [82]. Oxidative stress has also been considered, at least in part, a consequence of Ca^2+^ entry into cells following non-specific membrane permeabilization by pre-fibrillar aggregates. The latter can result from structural modifications of the membrane following the interaction with the aggregates or their monomers (see above), from membrane lipid peroxidation or from chemical modification of membrane ion pumps (reviewed in [4]), [141, 145]. Increased levels of intracellular free Ca^2+^ can stimulate the oxidative metabolism providing the ATP needed to support the activity of membrane ion pumps involved in clearing the excess Ca^2+^. The resulting ROS elevation would, in turn, oxidize membrane pumps and their regulatory proteins resulting in further free Ca^2+^ increase [146] with uncontrolled ingress of Ca^2+^ into, and release of pro-apoptotic signal from, the mitochondria. Such a chain of events, possibly occurring even in old age, could explain the relationship between ROS, intracellular free Ca^2+^ increase, mitochondrial damage and apoptosis described in cells exposed to toxic amyloid aggregates (reviewed in [4,81]), [85, 93, 147]. Recent data on the biochemical features possibly accounting for the different vulnerability of varying cell types exposed to the same toxic pre-fibrillar aggregates highlight significant correlations between cell resistance, cholesterol content, total antioxidant capacity and Ca^2+^-ATPase activity [112].

## 7. Conclusions

The work carried on in the last ten years has provided significant steps forward in the knowledge of how a polypeptide chain folds into the unique compact and biologically active protein structure. Increasing information exploiting new spectroscopic, imaging, computing and simulation techniques makes it likely that we are starting to unravel the protein folding code. This is expected to have important outcomes in many areas of genomics and structural biology, including a better knowledge of protein unfolding and aggregation. Actually, it has emerged that protein folding and protein ordered aggregation rely on the same physicochemical parameters thus stressing the key importance of the structural adaptations evolved in order to select amino acid sequences endowed with the lowest propensity to unfold and aggregate in the complex and crowded intracellular milieu. It also led us to consider that protein folding and aggregation are processes closely related with a shared energy landscape where different conformational states most often in equilibrium to each other can be populated.

Finally, the increased knowledge on the fundamentals of protein folding, misfolding and aggregation enables us to better understand the effects, on these, of external factors, such as temperature, pH, mutations, chemical modifications, molecular crowding and surfaces. In particular, the latter are increasingly recognised as important elements affecting remarkably the behaviour of a polypeptide chain providing it an environment with special physicochemical features, most often very different from those encountered in the bulk solution. Increasing data on the roles of surfaces in protein folding, misfolding and aggregation highlight contrasting effects. Some surfaces, such as those resulting from protein evolution, are able to promote protein folding over aggregation, as in the case of the molecular chaperones and the specific targets of the natively unstructured proteins. However, in other cases synthetic or biological surfaces can favour protein misfolding and aggregation over normal folding, as it is shown by a number of experimental results carried out on synthetic phospholipid membranes or SDS micelles, inorganic surfaces such as mica, or macromolecules such as glycosaminoglycans, collagen and nucleic acids. In some cases, these researches have shed light on the possible factors favouring the aggregation of specific proteins such as b2m and on the tissue specificity of the deposition of its aggregates.

In conclusion, it can be expected that the knowledge gained from protein folding and aggregation studies will give new insights into the nature of amyloid diseases and will help to provide a more rational basis for novel therapeutic strategies.
